# Which one came first: movement behavior or frailty? A cross‐lagged panel model in the Toledo Study for Healthy Aging

**DOI:** 10.1002/jcsm.12511

**Published:** 2020-01-08

**Authors:** Asier Mañas, Borja del Pozo‐Cruz, Irene Rodríguez‐Gómez, José Losa‐Reyna, Leocadio Rodríguez‐Mañas, Francisco J. García‐García, Ignacio Ara

**Affiliations:** ^1^ GENUD Toledo Research Group University of Castilla‐La Mancha Toledo Spain; ^2^ CIBER of Frailty and Healthy Aging (CIBERFES) Madrid Spain; ^3^ Motivation and Behaviour Research Program, Institute for Positive Psychology and Education, Faculty of Health Sciences Australian Catholic University Sydney NSW Australia; ^4^ Geriatric Department Hospital Virgen del Valle Toledo Spain; ^5^ Geriatric Department Hospital Universitario de Getafe Getafe Spain

**Keywords:** Structural equation modelling, Longitudinal, Exercise, Sedentary time, Ageing, Functioning and disability

## Abstract

**Background:**

There has been limited longitudinal assessment of the relationship between moderate‐to‐vigorous physical activity (MVPA) and sedentary behaviour (SB) with frailty, and no studies have explored the possibility of reverse causality. This study aimed to determine the potential bidirectionality of the relationship between accelerometer‐assessed MVPA, SB, and frailty over time in older adults.

**Methods:**

Participants were from the Toledo Study for Healthy Aging. We analysed 186 older people aged 67 to 90 (76.7 ± 3.9; 52.7% female participants) over a 4‐year period. Time spent in SB and MVPA was assessed by accelerometry. Frailty Trait Scale was used to determine frailty levels. A cross‐lagged panel model design was used to test the reciprocal relationships between MVPA/SB and frailty.

**Results:**

Frailty Trait Scale score changed from 35.4 to 43.8 points between the two times (*P* < 0.05). We also found a reduction of 7 min/day in the time spent on MVPA (*P* < 0.05), and participants tended to spend more time on SB (*P* = 0.076). Our analyses revealed that lower levels of initial MVPA predicted higher levels of later frailty [std. *β* = −0.126; confidence interval (CI) = −0.231, −0.021; *P* < 0.05], whereas initial spent time on SB did not predict later frailty (std. *β* = −0.049; CI = −0.185, 0.087; *P* = 0.48). Conversely, an initial increased frailty status predicted higher levels of later SB (std. *β* = 0.167; CI = 0.026, 0.307; *P* < 0.05) but not those of MVPA (std. *β* = 0.071; CI = −0.033, 0.175; *P* = 0.18).

**Conclusions:**

Our observations suggest that the relationship between MVPA/SB and frailty is unidirectional: individuals who spent less time on MVPA at baseline are more likely to increase their frailty score, and individuals who are more frail are more likely to spent more time on SB at follow‐up. Interventions and policies should aim to increase MVPA levels from earlier stages to promote successful aging.

## Introduction

Globally, the population aged 65 and over is growing faster than all other age groups^1^, ^2^. One of the most remarkable changes in body composition related to aging is the loss of skeletal muscle (i.e. sarcopenia).^3^ Frailty, defined as a condition of increased vulnerability associated with aging, and sarcopenia have been linked because both can lead to disability, hospitalization, and premature death.^4^, ^5^, ^6^ Sarcopenia has been considered both as the biological substrate for the development of physical frailty and the pathway through which adverse health‐related outcomes of physical frailty occur.^7^ Consequently, interventions to reduce the burden associated with frailty should be focused, among others, on skeletal muscle and its functionality.^8^

Increasing physical activity and reducing the levels of sedentary behaviour (SB) have been suggested to be a key strategy to attenuate the declines in muscle mass and physical function associated with aging and may also delay the clinical symptoms of frailty in older adults.^9^ Several cross‐sectional studies suggest that objectively assessed moderate‐to‐vigorous physical activity (MVPA) and SB are related with frailty in middle‐aged to older‐aged adults.^10^, ^11^, ^12^, ^13^ Nonetheless, a major limitation of the existing evidence is that it mainly relies on cross‐sectional designs, thus precluding us from making any causal inferences due to the inability of establishing the temporal sequence of the effects of MVPA/SB on frailty outcomes.

Therefore, longitudinal studies are essential because they provide an opportunity to explore in more detail the causal direction of the associations between MVPA/SB and frailty, knowledge which could then contribute to the development of intervention strategies to favour successful aging outcomes, including frailty and associated symptoms. To date, most of the existing longitudinal studies found a positive association between SB and frailty^14^, ^15^ and an inverse association for MVPA and frailty.^16^ For example, a longitudinal study in two Spanish cohorts of community‐dwelling older adults reported baseline television viewing time was also associated with frailty at 4‐year follow‐up.^17^ Nevertheless, all of these studies used self‐report methods to assess the movement behaviour of interest. Song et al.^18^ showed a relationship between objectively assessed sedentary time and development of physical frailty. However, an important caveat with this study is that used gait speed as a proxy measure of frailty thus could not capture the multidimensional nature of frailty.^4^ To our knowledge, there is no longitudinal study that has objectively measured both frailty and MVPA/SB.

The relationships between physical activity, sedentary time, and frailty are further complicated by the possibility of reverse causality.^16^, ^19^ In all previous longitudinal studies, whether using objective or subjective measures of the variables of interest, the authors investigated the prospective associations of MVPA/SB with frailty, not taking into account the potential reverse or temporal order in the causality chain. We cannot rule out the possibility that a high level of frailty can be associated with lower levels of physical activity and a greater amount of sedentary time at a future time. The reverse may also be true. Estimating the temporal ordering, and potential bidirectionality of the association of SB and MVPA with frailty would be advantageous to inform subsequent interventions aimed at reducing the burden of frailty among the older population. However, no studies exist investigating this issue.

With fill in this gap by examining the longitudinal association of accelerometer‐assessed MVPA and SB with frailty over a period of 4 years in a population sample of older adults from the Toledo Study for Healthy Aging (TSHA). In doing so, we applied a cross‐lagged panel model, a statistical technique appropriate for the context and aims of the current study.^20^, ^21^ Specifically, this study examined whether MVPA/SB predicted frailty in the future and whether frailty predicted subsequent movement behaviours (or both). As far as we know, this is the first study that has examined the potentially reciprocal relationships between movement behaviours and frailty in older adults. In the current study, four hypotheses were tested:

H_0_: Frailty does not predict changes in SB/MVPA, and SB/MVPA does not predict changes in frailty.

H_1_: Frailty predicts changes in SB/MVPA, but SB/MVPA does not predict changes in frailty.

H_2_: SB/MVPA predicts changes in frailty, but frailty does not predict changes in SB/MVPA.

H_3_: SB/MVPA and frailty have a reciprocal relationship—frailty predicts changes in SB/MVPA, and SB/MVPA predicts changes in frailty.

## Methods

### Study design and participants

**Figure 1:**
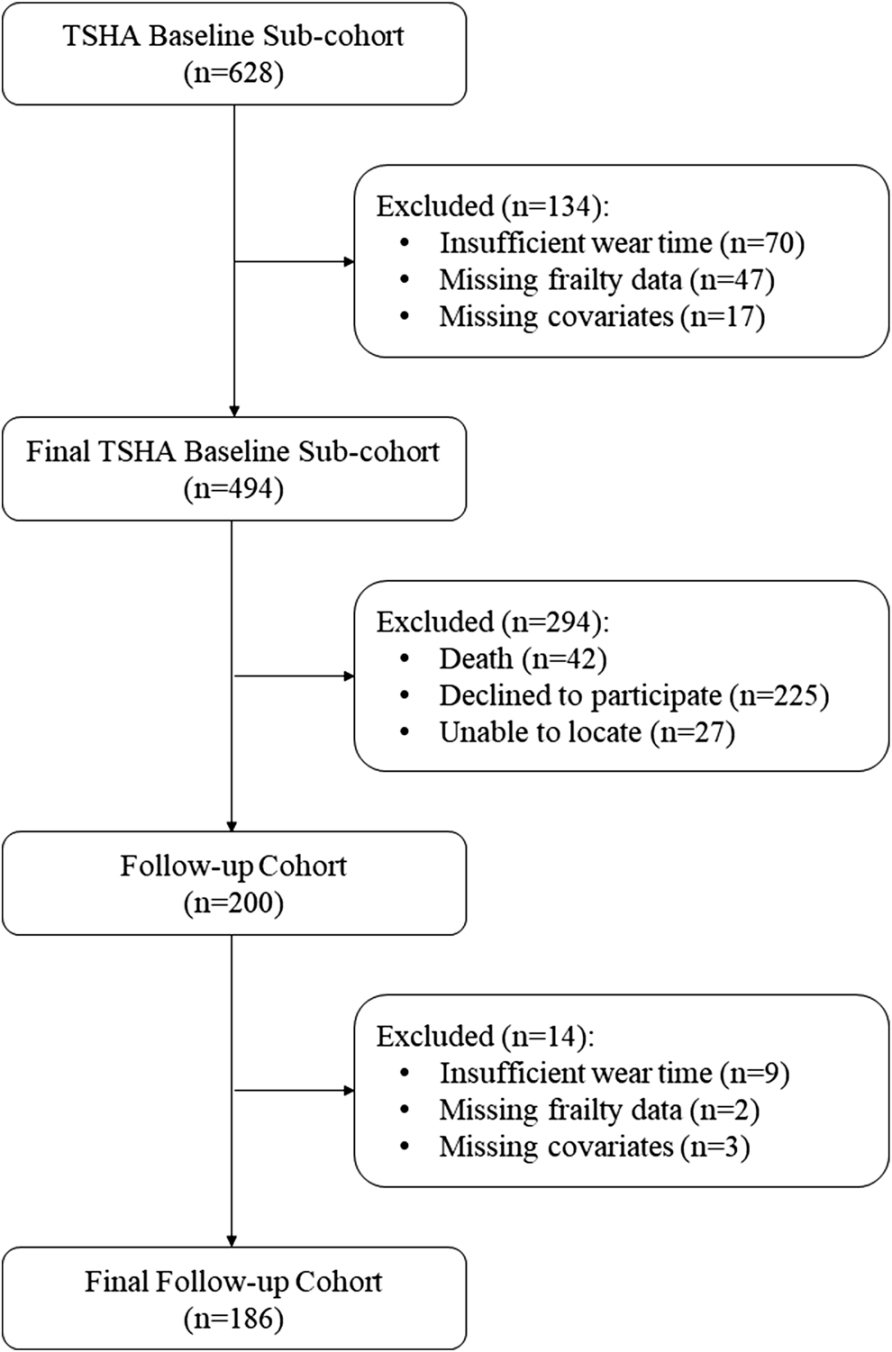
Flow diagram of the process for obtaining the final sample of the study.

This is a longitudinal study consisting of two data collection waves separated by 4 years (3.8 ± 0.8 years). This investigation used data from the second and third waves of the TSHA. Details of the protocol of the TSHA are described elsewhere.^22^, ^23^ Briefly, the TSHA is a population prospective cohort study aimed at studying the determinants and consequences of frailty in institutionalized and community‐dwelling individuals older than 65 years living in the province of Toledo, Spain. In the current study, a subsample of the TSHA with accelerometer data was included. A total of 277 men and 351 women over 65 years of age at baseline, although 494 participants, concluded the three stages of assessment and provide with valid data for the analyses [224 men (45.3%)]. The first time point of assessment for this study started in July 2012 and lasted until June 2014. In the first stage, six psychologists conducted computer‐assisted interviews face to face with potential subjects. In the second stage, three nurses performed a physical examination followed by clinical and performance tests at the subject's home. In the third stage, the participants were invited to wear an accelerometer for a week. Participants were contacted again in 2015 and invited to participate in a follow‐up study conducted between May 2015 and July 2017.^24^ After the follow‐up, 200 participants (59.5% missing) completed the second evaluation. However, 186 subjects [88 men (47.3%) with complete data on all exposures, outcomes, and ≥80% covariates] were included in the final analyses of this study (see *Figure* 1 for the study participant flow diagram). Signed informed consent was obtained from all participants prior participation in the study. The study was approved by the Clinical Research Ethics Committee of the Toledo Hospital, which was conducted according to the ethical standards defined in the 1964 Declaration of Helsinki.

### Measurements

#### Frailty status

The Frailty Trait Scale (FTS)^25^ was used to assess frailty in this study. The FTS includes seven aspects: energy balance and nutrition, activity, nervous system, vascular system, weakness, endurance, and slowness. These domains become operational through 12 items:

- Body mass index (BMI), central obesity (waist circumference), unintentional weight loss, and serum albumin level were used to assess energy balance and nutrition.
- Activity was assessed using the total score of the Physical Activity Scale for the Elderly.^26^
- The nervous system was calculated by considering verbal fluency and balance. Verbal fluency was estimated by asking the participants to give names of animals during 1 min.^27^
- Balance was measured by Romberg test.^28^
- The vascular system was measured by the brachial‐ankle index performed with Doppler ultrasound.^29^
- Weakness was estimated assessing grip strength in the dominant arm and the knee extension strength.^23^
- Endurance was assessed by the chair stand test, which measures the number of times that a person stands up in 30 s.^30^
- Slowness was estimated by calculating the time to walk 3 m at a ‘normal pace’ according to a standard protocol.^28^

Each item score represents a biological trait and ranges from 0 (*the best*) to 4 (*the worst*), except in the ‘chair test’ where the range is from 0 to 5 points because of the necessity of scoring those unable to stand a single time. When appropriate, items are analysed according to the item's quintile distribution in the population.

To be included in the study, the participants had to overcome at least 75% (9 of the 12) of the items included in the FTS.^25^ The total score was calculated by adding all the scores in each item divided by total score for each individual and multiplying by 100, standardizing the measure to a range from 0 (*best score*) to 100 (*worst score*), according to the formula total score = (Σ items score/total score possible by individual)*100.

#### Physical activity and sedentary behaviour assessment

The ActiGraph accelerometer ActiTrainer and wGT3X‐BT (ActiGraph, LLC, Pensacola, FL) were used to assess the participants' physical activity and SB levels during a week as previously described.^11^ In brief, participants were instructed to wear an accelerometer on the left hip during waking hours, with exception for water activities. The devices were initialized to collect data using 1‐min epochs, and all data were collected using the vertical axis collection mode. Inclusion criteria comprised at least 4 days with at least 8 h recorded per day without excessive counts (i.e. >20,000 counts).^31^ Non‐wear time was defined as a minimum of 60 min with allowance of 1–2 min of counts below 100 counts.^32^ Daily average times spent in SB (<100 counts/min) and MVPA (≥1952 counts/min) were derived according to previous work.^33^ Although there is a lack of consensus on the use of cut‐off points to classify the intensity of the activity, the cut‐off points used in this study are the most commonly reported in this population group,^34^ and this makes our results comparable with other studies. Minutes spent in each of these three behaviours were tallied per day and averaged over all available valid days.

#### Anthropometrics and confounding variables

Height was measured to the nearest centimetre using a stadiometer (Seca 711 scales, Hamburg, Germany), and weight was measured with a Seca precision scale (Seca 711 scales, Hamburg, Germany). Individuals removed their shoes, socks, and heavy clothes prior to weighing. BMI was calculated as weight (kg) divided by height squared (m^2^).

Participants self‐reported their age, sex, and ethnicity. Education (no studies, primary school completed, secondary school completed, or more), marital status (single, married/living together, widowed, and divorced/separated), and income (it was coded into three categories ranging from any income to 3000€ per month) were also self‐reported in face‐to‐face interviews. We also evaluated objective cognitive function using the mini‐mental state examination.^35^

#### Statistical analysis

Preliminary analyses examined variable distributions, sample characteristics, and attrition using R software (R project version 3.5.1). Descriptive variables were compared between participants retained with those of participants not retained from wave1 to wave2 with an independent *t*‐test or χ^2^ test for continuous and categorical variables, respectively. Descriptive statistics [mean and standard deviation (SD) for continuous variables and as frequencies and percentages for categorical variables] were calculated for all outcome measurements. Comparison between baseline and follow‐up time continuous variables was performed using a paired sample *t*‐test.

We tested our hypotheses using structural equation modelling with maximum likelihood estimation using functions from the R package lavaan.^36^ Full information maximum likelihood was used to provide unbiased and efficient estimates of the parameters of interest missingness at random.^37^ Two cross‐lagged panel models were used to test the hypothesis of the study. A cross‐lagged panel model was implemented to test the relationships between SB and frailty status across the two time points for the present study (i.e. initial assessment and 4‐year follow‐up). The second cross‐lagged panel model was used to test the relationships between MVPA and frailty status. The null hypotheses would be supported if neither of the coefficients associated with the cross paths were significantly different from zero. If the cross path towards frailty in time 2, but not towards SB/MVPA in time 2, was significant, then H_1_ would be supported. If it were the reverse of the latter, then H_2_ would be supported. Finally, if both paths were significant, then H_3_ would be supported. Analyses included sex as time‐invariant variable; in addition, age, education, marital status, income, BMI, mini‐mental state examination, and accelerometer wear time were allowed to be time‐varying covariates (i.e. allowing for possible changes in these measures from initial assessment to follow‐up). Among the strengths of using a cross‐lagged panel approach is that it allows simultaneous analysis of the two dependent outcomes, thereby permitting the identification of possible bidirectional associations over time. Model fit was assessed using a selection of fit indices and criteria: root mean square error of approximation (RMSEA) (≤0.06), standardized root mean square residual (SRMR) (≤0.08), confirmatory fit index (CFI) (≥0.95), and Tucker‐Lewis index (TLI) (≥0.95).^38^

## Results

### Attrition/missing data across time points

Participants decreased from 494 with complete data at baseline to 186 with complete data at follow‐up assessment (see Figure 1). The causes and numbers who were lost to the follow‐up assessment were death (*n* = 42), refusal (*n* = 225), and could not be located (*n* = 27). Additional missing data were lost by insufficient accelerometer wear time data (*n* = 9), missing frailty data (*n* = 2), or losing more than 80% of the covariates (*n* = 3). Compared with the retained sample, participants who dropped the study at 4‐year follow‐up were significantly older, less educated, and spent more time on SB (Data S1). Also, MVPA had a trend toward significance reductions in those participants. Missing data were addressed using a full information maximum likelihood algorithm, as recommended elsewhere.^39^

### Descriptive statistics

Means and standard deviations for MVPA, SB, and frailty as well as confounders at each of the two time‐points of assessment (viz. T1 and T2) for the present study are shown in Table 1. At baseline, participants had a mean age of 76.68 (SD = 3.90), a mean FTS of 35.35 (SD = 13.94), a mean time (min/day) spent on SB of 530.18 (SD = 84.86), and an MVPA of 20.12 (SD = 23.30). FTS score increased significantly between T1 and T2 (*P* < 0.05). It was found that participants tended to spend more time on SB (*P* = 0.076), and there was a significant reduction in the time spent on MVPA between the two times (*P* < 0.05). BMI and MSSE also decreased significantly at both time points (*P* < 0.05).

**Table 1:** Sociodemographic and descriptive data.

Variables	Baseline	Follow‐up
	(n=186)	(n=186)
Age (years)^a^	76.68 ± 3.90	80.44 ± 4.24*
Sex^b^		
Men	88 (47.3)	88 (47.3)
Women	98 (52.7)	98 (52.7)
BMI (kg/m^2^)^a^	30.82 ± 4.62	30.33 ± 4.40*
Education^b^		
None	139 (74.7)	116 (62.4)
Primary school	30 (16.1)	51 (27.4)
Secundary or more	14 (7.5)	19 (10.2)
Missing^c^	3 (1.6)	−
Income^b^		
Low	87 (46.8)	75 (40.4)
Medium	87 (46.8)	70 (37.6)
High	9 (4.8)	6 (3.2)
Missing^c^	3 (1.6)	35 (18.8)
Marital status^b^		
Single	7 (3.8)	7 (3.8)
Married	136 (73.1)	125 (67.2)
Widower	40 (21.5)	51 (27.4)
Separated/Divorced	1 (0.5)	2 (1.1)
Missing^c^	2 (1.1)	1 (0.5)
MSSE^a^	24.02 ± 3.73	23.32 ± 3.54*
Missing^c^	15 (8.1)	15 (8.1)
Frailty Trait Scale, points^a^	35.35 ± 13.94	43.79 ± 13.86*
Accelerometer wear time, min/valid day^a^	781.36 ± 83.14	777.61 ± 74.45
Sedentary time, min/valid day^a^	530.18 ± 84.86	542.61 ± 75.91Ŧ
MVPA, min/valid day^a^	20.12 ± 23.30	13.21 ± 18.73*

Abbreviations: BMI, body mass index; MSSE, mini‐mental scale examination; MVPA, moderate‐to‐vigorous physical activity.

a Continuous variable; mean standard ± deviation.

b Categorical variable; n (%).

c Missing data; n (%).

* Significant differences between baseline vs. follow‐up (*p*<0.05).

Ŧ Trend toward significance between baseline vs. follow‐up (*p*<0.08>0.05)

### Cross‐lagged panel model 1: moderate‐to‐vigorous physical activity

**Figure 2:**
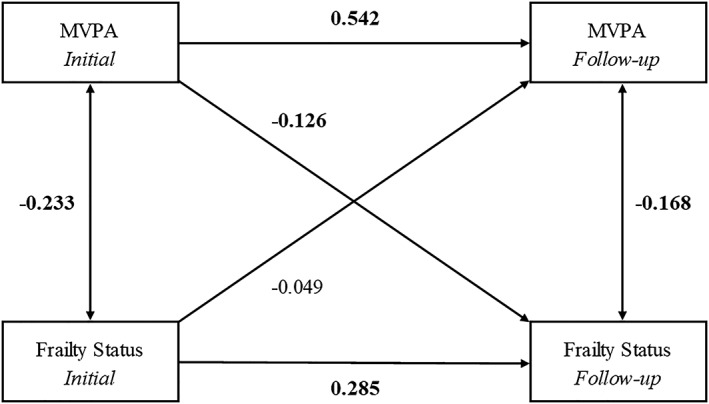
Cross‐lagged panel model 1: moderate‐to‐vigorous physical activity. MVPA, moderate‐to‐vigorous physical activity. Model adjusted for age, sex, body mass index, education, income, marital status, and mini‐mental scale examination. Bold indicates statistical significance (*P* < 0.05).

Figure 2 shows the final cross‐lagged model. The data fit the model well (RMSEA = 0.000; SRMR = 0.013; CFI = 1.000; TLI = 1.023). The largest effects on T2 MVPA and T2 frailty were those determined by the autoregressive pathways. That is, past MVPA and frailty scores predicted future MVPA and frailty scores, respectively. The cross‐lagged effect from MVPA at T1 to frailty status at T2 was statistically significant (standardized regression coefficient of −0.126; 95% CI = −0.231, −0.021; *P* < 0.05), indicating that higher levels of MVPA at baseline predicted lower frailty score 4 years later, adjusting for baseline frailty status. In contrast, the cross‐lagged effect from frailty status to MVPA was not statistically significant (standardized regression coefficient of −0.049; 95% CI = −0.185, 0.087; *P* = 0.48), suggesting that frailty did not predict future levels of MVPA.

### Cross‐lagged panel model 2: sedentary behaviour

**Figure 3:**
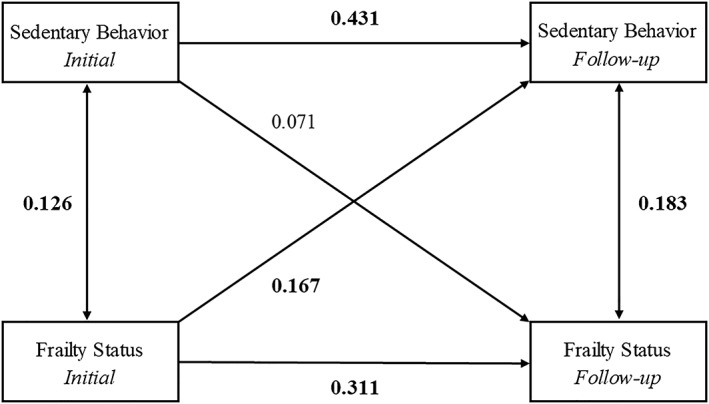
Cross‐lagged panel model 2: sedentary behaviour. Model adjusted for age, sex, body mass index, education, income, marital status, and mini‐mental scale examination. Bold indicates statistical significance (*P* < 0.05).

Figure 3 shows the final cross‐lagged model. The data fit the model well (RMSEA = 0.012; SRMR = 0.018; CFI = 0.997; TLI = 0.992). Similar to the previous model, the autoregressive SB and frailty pathways were statistically significant. The cross‐lagged effect from frailty to SB levels was statistically significant (standardized regression coefficient of 0.167; 95% CI = 0.026, 0.307; *P* < 0.05), indicating that higher levels of frailty at baseline predicted higher SB 4 years later, adjusting for baseline SB. In contrast, the cross‐lagged effect from SB levels to frailty was not statistically significant (standardized regression coefficient of 0.071; 95% CI = −0.033, 0.175; *P* = 0.18), suggesting that SB levels did not predict future frailty status.

## Discussion

The present study investigated the longitudinal relationships between MVPA and SB with frailty status in a community‐based sample of older adults. As a novelty, we applied a cross‐lagged panel model to test for potential reciprocal relationships between MVPA/SB and frailty over a 4‐year period. The main finding in our study was that accelerometer‐assessed initial MVPA predicted frailty score at follow‐up. However, baseline sedentary time was not significantly related to frailty after the follow‐up. We further found that initial frailty status predicted subsequent sedentary time (i.e. more frailty status was related to posterior higher levels of SB), but not of MVPA. These results have the potential to inform future interventions that aim at reducing the burden associated with frailty among older adults.

Different cross‐sectional^10^, ^11^, ^40^ and longitudinal^16^ studies have linked MVPA with frailty. Blodgett et al.^10^ found that MVPA was associated with frailty in a group of community dwelling adults aged over 50 from the National Health and Nutrition Examination Survey. Other longitudinal studies such as Rogers et al.^16^ have also confirmed these results in 8649 adults aged 50 and over an average of 10 years of follow‐up. We also found that MVPA prospectively predicted frailty levels in our sample. There are numerous arguments supporting these findings.^41^, ^42^, ^43^, ^44^ It has been demonstrated that physical activity, particularly of moderate intensity, plays an important role on multiple components of the frailty syndrome including the frailty phenotype, physiologic dysregulation, and cellular function.^9^ Increases in MVPA seem to also preserve or even improve muscle function and structure, protein synthesis, glucose metabolism, or inflammation.^43^ Furthermore, regular physical activity can maintain a set of bioenergetically functional mitochondria that, by improving systemic mitochondrial function, contribute to reducing the risk of morbidity and mortality throughout life.^41^ Not surprisingly, MVPA is considered a cornerstone for the prevention, delay, or treatment of frailty among older adults.

On the other hand, our results did not support the hypothesis that initial frailty levels predict future MVPA levels. Several studies support the predictive ability of physical functioning on subsequent MVPA levels.^45^, ^46^, ^47^ However, it may also be plausible that other non‐biological mechanisms (e.g. behavioural) accounted for the observations of the current study. Different intervention studies have shown the possibility of increasing physical activity also in frail participants.^48^ For example, Yamada et al.^49^ found that is possible to promote exercise of moderate‐to‐vigorous intensity among very frail older adults. Future studies are warranted to clarify the role of frailty in subsequent MVPA levels in older adults.

SB has recently been considered as an important factor for numerous health outcomes.^50^, ^51^ A recent systematic review has shown that SB may be associated with increased levels of frailty, particularly among the most vulnerable population.^52^ Interestingly, our results indicate that SB was not a determinant of frailty but rather a consequence of an altered state of increased frailty. A smaller study by Edholm et al.^53^ with 60 older woman found that only activities of at least moderate intensity were associated with physical function in a subsequent follow‐up time but not activities of lighter intensity or sedentary activities. In addition, Marques et al.^54^ in a study conducted with 131 male and 240 female participants aged 65–103 years, suggested that sedentary time was not a significant predictor of loss of physical independence in later life. In a previous cross‐sectional study, we showed that engaging in high levels of MVPA (i.e. 27 min/day) could cancel out the detrimental effects of SB on frailty, which may partly explain our current observations.^55^ Given that the relationship between SB and frailty may go beyond total accumulated time,^12^ future studies should enquire whether or not the results of this and other studies are confirmed for different patterns of accumulation of SB.

According to our findings, the promotion of MVPA at earlier stages will translate into more MVPA and less frailty markers in the future. Also, the observations of the current study point out to the possibility that the detrimental effects on frailty are primarily defined by insufficient amounts of MVPA rather than an excessive amount of sedentary time. Public health organizations should target MVPA to reduce the burden associated with frailty in older adults.

### Strengths and limitations

An important strength of our study is that it includes a relatively large sample of community‐dwelling older adults with longitudinal data separated by 4 years. It also includes accelerometer‐derived sedentary and physical activity behaviour estimations. Also, although there is no established gold standard to identify frailty, the FTS has been suggested as a measurement of frailty with superior predictive validity than previously validated scales such as the frailty phenotype^56^ and the frailty index.^57^ Additionally, a key strength of this study was that the statistical analysis deployed has allowed to explore the auto‐regressive and cross‐lagged pathways in exploring how frailty relates over time with both MVPA and SB. Despite the methodological rigour of this study, some limitations have to be acknowledged. First, we cannot rule out the possibility that our estimations could be influenced by the characteristics of the participants who did not provide valid data at follow‐up (i.e. older, less active, more sedentary, and less educated), and therefore, our results should be interpreted with caution. A further limitation of our work was that despite validity and widely used of accelerometers to assess physical activity in free living conditions, these devices are not able to discriminate between sitting and standing^58^ or activity type (e.g. running vs. muscle strength), which could potentially bias the estimations in our study. Finally, physical activity and SB have been examined separately from other lifestyle behaviours (e.g. diet, smoking, and alcohol consumption). However, lifestyle behaviours tend to cluster together. Therefore, it could be that our results rather reflect the synergistic consequences of different observed (i.e. physical activity and SB) and unobserved (e.g. diet quality) lifestyle behaviours.^59^, ^60^ Future studies may want to test this hypothesis.

## Conclusion

In summary, our findings suggest that MVPA, but not SB, predicts frailty in older adults. In contrast, frailty seems to be a predictor of SB but not of MVPA. Efforts should be directed at increasing MVPA from earlier stages. Future experimental studies should examine the best strategies to include MVPA in the daily lives of older people.^61^

## Conflict of interest

None to declared.

## Funding

This work was supported by the Biomedical Research Networking Center on Frailty and Healthy Aging (CIBERFES) and FEDER funds from the European Union (CB16/10/00477), (CB16/10/00456), and (CB16/10/00464). It was further funded by grants from the Government of Castilla‐La Mancha (PI2010/020), Institute of Health Sciences, Ministry of Health of Castilla‐La Mancha (03031‐00), Spanish Government [Spanish Ministry of Economy, ‘Ministerio de Economía y Competitividad’, and Instituto de Salud Carlos III (PI10/01532, PI031558, and PI11/01068)], and by European Grants (Seventh Framework Programme: FRAILOMIC). Asier Mañas Bote received a PhD grant from the Universidad de Castilla‐La Mancha ‘Contratos predoctorales para la formación de personal investigador en el marco del Plan Propio de I+D+i, cofinanciados por el Fondo Social Europeo’ (2015/4062).

## Supporting information

Data S1. Comparison of characteristics at baseline of participants retained with those of participants not retained from wave1‐wave2.
