# Supplementary material for: Which one came first: movement behavior or frailty? A cross‐lagged panel model in the Toledo Study for Healthy Aging
Source: J Cachexia Sarcopenia Muscle. 2020 Jan 8;11(2):415–23. doi: 10.1002/jcsm.12511 (PMC7113532; doi:10.1002/jcsm.12511)
Supplement: Supplementary file 1 — Data S1. Comparison of characteristics at baseline of participants retained with those of participants not retained from wave1‐wave2. [file JCSM-11-415-s001.docx]

**Supplementary File 1.** Comparison of characteristics at baseline of participants retained with those of participants not retained from wave1-wave2.

|  |  | | |  | | |  |  |  |  |  |
| --- | --- | --- | --- | --- | --- | --- | --- | --- | --- | --- | --- |
| Variables | Retained | | | Not retained | | | *P* value |  |  |  |  |
|  | (n=186) | | | (n=308) | | |  |  |  |  |  |
| Age (years)^a^ | 76.68 ± 3.90 | | | 78.17 ± 4.69 | | | **0.003** |  |  |  |  |
| Sex^b^ |  | | |  | | | 0.495 |  |  |  |  |
| Men | 88 (47.3) | | | 137 (44.2) | | |  |  |  |  |  |
| Women | 98 (52.7) | | | 173 (55.8) | | |  |  |  |  |  |
| BMI (kg/m^2^)^a^ | 30.82 ± 4.62 | | | 30.48 ± 4.82 | | | 0.497 |  |  |  |  |
| Education^b^ |  | | |  |  |  | **0.014** |  |  |  |  |
| None | 139 (74.7) | | | 262 (84.5) | | |  |  |  |  |  |
| Primary school | 30 (16.1) | | | 25 (8.1) | | |  |  |  |  |  |
| Secundary or more | 14 (7.5) | | | 19 (6.1) | | |  |  |  |  |  |
| Missing^c^ | 3 (1.6) | | | 4 (1.3) | | |  |  |  |  |  |
| Income^b^ |  | | |  | | | 0.888 |  |  |  |  |
| Low | 87 (46.8) | | | 136 (43.9) | | |  |  |  |  |  |
| Medium | 87 (46.8) | | | 166 (44.2) | | |  |  |  |  |  |
| High | 9 (4.8) | | | 21 (6.8) | | |  |  |  |  |  |
| Missing^c^ | 3 (1.6) | | | 16 (5.2) | | |  |  |  |  |  |
| Marital status^b^ |  |  |  |  |  |  | 0.579 |  |  |  |  |
| Single | 7 (3.8) | | | 18 (5.8) | | |  |  |  |  |  |
| Married | 136 (73.1) | | | 211 (68.1) | | |  |  |  |  |  |
| Widower | 40 (21.5) | | | 76 (24.5) | | |  |  |  |  |  |
| Separated/Divorced | 1 (0.5) | | | 1 (0.3) | | |  |  |  |  |  |
| Missing^c^ | 2 (1.1) | | | 4 (1.3) | | |  |  |  |  |  |
| MSSE^a^ | 24.02 ± 3.73 | | | 22.90 ± 4.82 | | | 0.207 |  |  |  |  |
| Missing^c^ | 15 (8.1) | | | 36 (11.7) | | |  |  |  |  |  |
| Frailty Trait Scale, points^a^ | 35.35 ± 13.94 | | | 40.53 ± 14.32 | | | 0.093 |  |  |  |  |
| Accelerometer wear time, min/valid day^a^ | 781.36 ± 83.14 | | | 781.76 ± 86.86 | | | 0.693 |  |  |  |  |
| Sedentary time, min/valid day^a^ | 530.18 ± 84.86 | | | 542.79 ± 98.05 | | | **0.043** |  |  |  |  |
| MVPA, min/valid day^a^ | 20.12 ± 23.30 | | | 16.71 ± 21.19 | | | *0.055* |  |  |  |  |
|  |  | | |  | | |  |  |  |  |  |

Abbreviations: BMI, body mass index; MSSE, mini-mental scale examination; MVPA, moderate-to-vigorous physical activity.

^a^Continuous variable; mean (standard deviation).

^b^Categorical variable; n (%).

^c^Missing data; n (%).

**Bold** indicates statistical significance (*p*<0.05) and *Italics* a trend toward significance (*p*<0.08>0.05).
